# Trio Clinical Exome Sequencing in a Patient With Multicentric Carpotarsal Osteolysis Syndrome: First Case Report in the Balkans

**DOI:** 10.3389/fgene.2018.00113

**Published:** 2018-04-05

**Authors:** Aleksandra Stajkovska, Sanja Mehandziska, Margarita Stavrevska, Kristina Jakovleva, Natasha Nikchevska, Zan Mitrev, Ivan Kungulovski, Gjorgje Zafiroski, Velibor Tasic, Goran Kungulovski

**Affiliations:** ^1^Bio Engineering LLC, Skopje, Macedonia; ^2^Zan Mitrev Clinic, Skopje, Macedonia; ^3^Neuromedica Hospital, Skopje, Macedonia; ^4^Department of Pediatric Nephrology, Medical Faculty of Skopje, University Children's Hospital, Skopje, Macedonia

**Keywords:** next-generation sequencing, exome sequencing, case report, multicentric carpotarsal osteolysis syndrome, Balkan

## Abstract

Exome sequencing can interrogate thousands of genes simultaneously and it is becoming a first line diagnostic tool in genomic medicine. Herein, we applied trio clinical exome sequencing (CES) in a patient presenting with undiagnosed skeletal disorder, minor facial abnormalities, and kidney hypoplasia; her parents were asymptomatic. Testing the proband and her parents led to the identification of a *de novo* mutation c.188C>T (p.Pro63Leu) in the *MAFB* gene, which is known to cause multicentric carpotarsal osteolysis syndrome (MCTO). The c.188C>T mutation lies in a hotspot amino acid stretch within the transactivation domain of MAFB, which is a negative regulator of RANKL-induced osteoclastogenesis. MCTO is an extremely rare autosomal dominant (AD) disorder that typically arises spontaneously and causes carpotarsal osteolysis, often followed by nephropathy. To the best of our knowledge, this is the first study reporting genetically diagnosed MCTO in the Balkans.

## Case presentation

**Figure 1:**
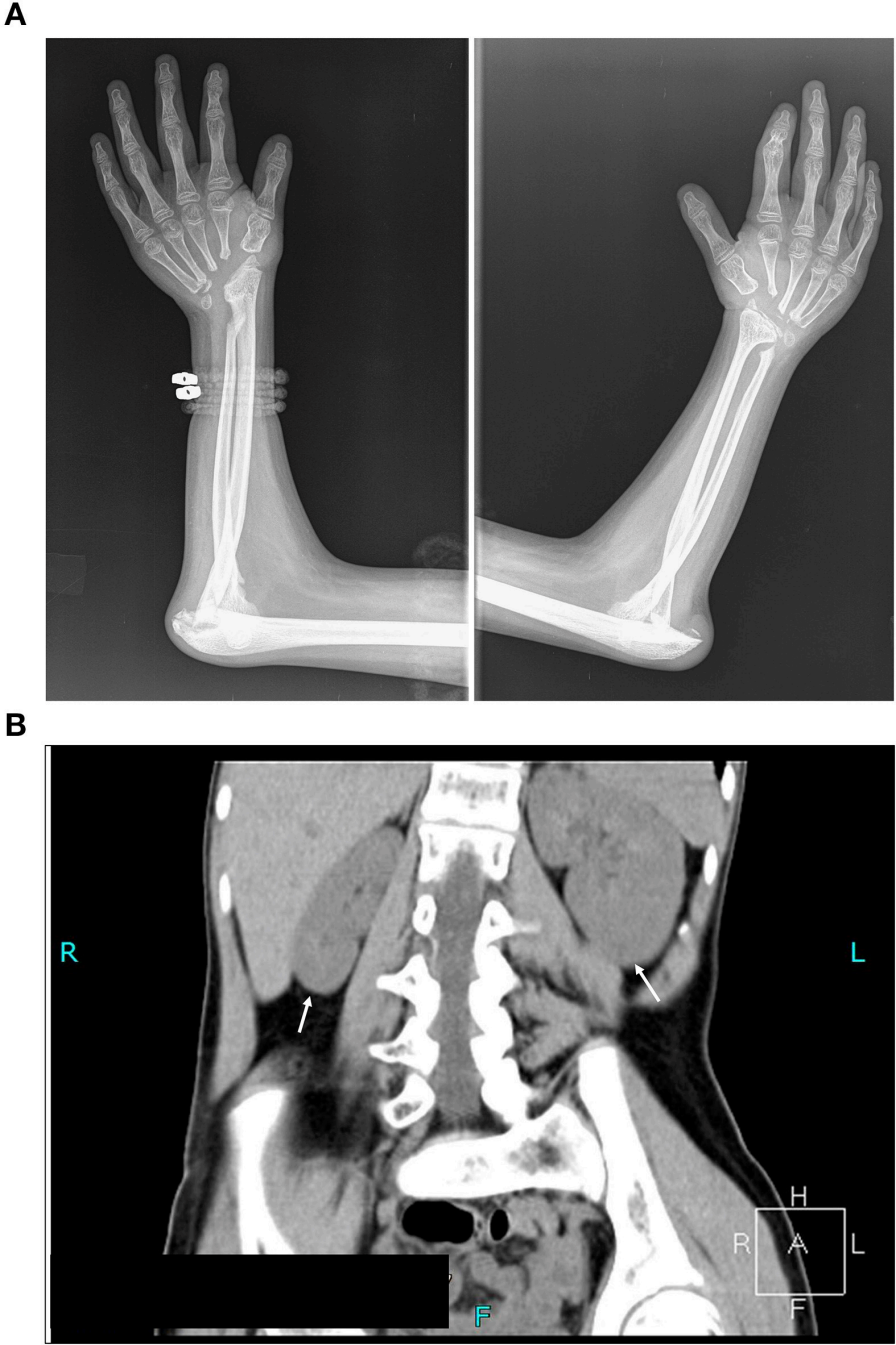
Assessment of the clinical symptoms of the patient. **(A)** X-ray image illustrating destruction of carpal bones. Absence of carpal bones and osteolysis of proximal end of metacarpal bones, and bones of the elbow joints with pathological dislocation of both elbow joints **(B)** Computed tomography scan (CT) illustrating kidney asymmetry. Please note the hypoplasia of the right kidney, and compensatory hypertrophy of the left kidney, and scoliosis dextroconvexa lumbalis and spina bifida of the lumbosacral vertebra (see also Supplementary Figure 1B).

A 13-year-old ethnic Macedonian girl and her parents attended the Zan Mitrev Clinic for pediatric examination, and concomitant genetic counseling. She presented with abnormal gait due to decreased joint mobility. Further investigations revealed destruction of carpal bones on both hands, olecranon bursitis, marfanoid habitus, cachexia, cutis laxa, mild facial abnormalities (triangular face, eye bulging, micrognathia). She did not show signs of intellectual disability; morphological abnormalities were absent in her parents (Figure 1; Supplementary Figures 1, 2).

The symptoms were first noted on her wrists and ankles when she was a toddler. At 13 years of age, her radiological examination showed osteolysis of carpal bones, destruction of the elbow joints with pathological dislocation of both elbows, osteolysis of the tarsal bones and first metatarsal with pathological dislocation of the first metatarsophalangeal joints, and cyst-like structures on the right femoral epiphysis (Figure 1A; Supplementary Figures 1, 2). Previous nephrology evaluation via ultrasound scanning revealed asymmetry in the size of the kidneys at 2 years of age. At that time, Tc^99^mDMSA scan showed 33% decreased uptake of the right kidney, however her blood work including renal function parameters were within reference limits. A recent ultrasound examination confirmed significant kidney hypoplasia of the right kidney (59 × 25 mm), in contrast, the left kidney was compensatorily hypertrophic with dimensions 107 × 45 mm. Computed tomography (CT) scan confirmed the ultrasound findings (Figure 1B). No audiometric and ophthalmological tests were carried out.

Upon taking written and signed informed consent from the proband's legal guardians, she underwent clinical exome sequencing (CES) (TruSight One panel) from Illumina. In brief, DNA was extracted from 400 μl of whole blood on a SaMag-12 automatic nucleic acid extraction system (Sacace Biotechnologies, Como, Italy). Libraries were pooled together and underwent two rounds of hybridization and capture, and additional quantification and size assessment. Between 19 and 26 million reads were obtained with a NextSeq machine (Illumina, San Diego, USA), with a coverage of at least 50x for 80–90% of all sequences. Sequence quality control was done with FastQC (https://www.bioinformatics.babraham.ac.uk/projects/fastqc/), and sequences were mapped to Hg19 with BWA (Li and Durbin, 2009). SNP and INDEL calling, together with advanced variant annotation were done with proprietary technologies such as PEPPER™ and MOKA™, respectively, both integrated within the Sophia DDM platform (Sophia Genetics, Saint-Sulpice, Switzerland). Later on, mapping, SNP and INDEL calling, annotation, and in-depth analyses were carried out with the Genoox platform (https://www.genoox.com/).

The analysis focused on virtual gene panels composed of genes causing skeletal dysplasia, and connective tissue disorders using the Sophia DDM platform (Sophia Genetics). The analysis failed to identify known Clinvar mutations. We detected several (>10) heterozygous candidate variants based on ACMG computational criteria (Richards et al., 2015; Supplementary Table 1).

**Figure 2:**
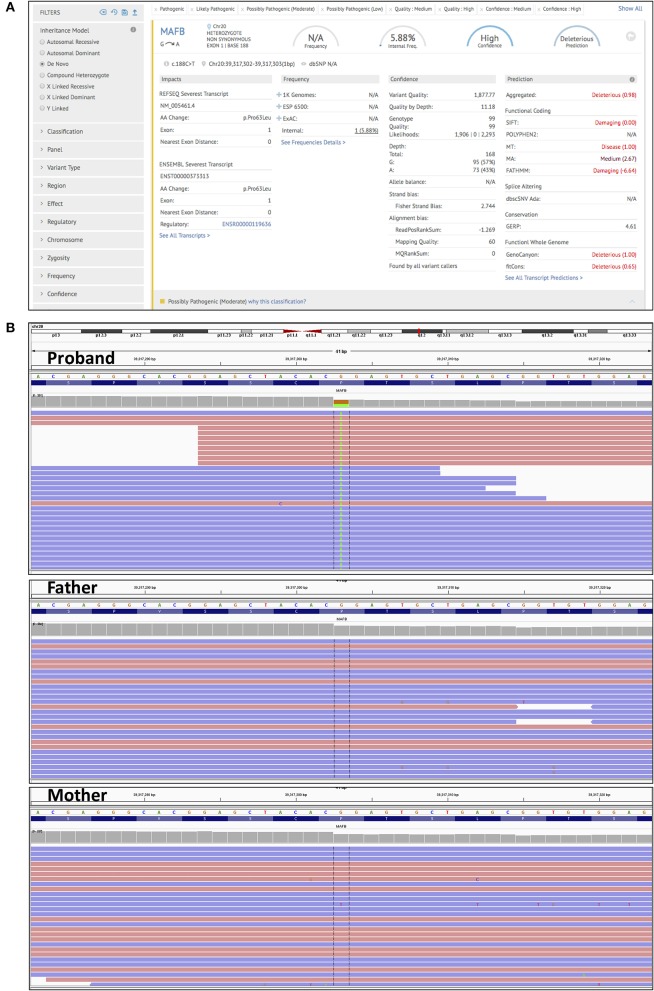
Trio clinical exome sequencing. **(A)** A screen shot taken from the Genoox platform, showing the detection and annotation of *de novo* c.188C>T (p.Pro63Leu) mutation in the *MAFB* gene. **(B)** IGV genome browser screenshot of reads obtained by NGS. Detection of the c.188C>T (p.Pro63Leu) in the *MAFB* gene in the proband (upper panel), and its absence in the father, and mother (middle and lower panel).

In order to accurately pin down the causal variant, we performed additional CES of her parents. Upon analysis of high confidence *de novo* mutations (present in the proband, but absent in the parents), using an in-built tool in the Genoox platform we identified the c.188C>T (p.Pro63Leu) variant in the *MAFB* gene (Figure 2; Supplementary Table 2). This finding was confirmed by Sanger sequencing (Supplementary Figure 3). This variant has been reported to cause multicentric carpotarsal osteolysis syndrome (MCTO), OMIM # 166300, (Zankl et al., 2012; Mehawej et al., 2013). The mutation lies in a hotspot amino acid stretch within the transactivation domain of MAFB, which is a negative regulator of RANKL-induced osteoclastogenesis (Mumm et al., 2014). The mutation also meets computational criteria of pathogenicity such as low population frequency (g1000, ExAC), damaging effect (SIFT, Mutation Taster, FATHMM), and evolutionary conservation (GERP) (Supplementary Table 2).

Next, we focused our attention to additional two heterozygous genetic variants as well. The first one, c.1851C>A (p.Asp617Glu) in the *COL9A3* gene is a variant of unknown significance (VUS) and it was detected in the proband and her father. The second one, c.908C>T (p.Thr303Met) in the *MATN3* gene was detected in the proband and her mother; it has been reported to be associated with osteoarthritis (Supplementary Table 1; Stefánsson et al., 2003; Pullig et al., 2007). The COL9A3 and MATN3 proteins are known to co-interact in their native state (Fresquet et al., 2007). In addition, known pathogenic variants in the *COL9A3* and *MATN3* genes have been reported to cause multiple epiphyseal dysplasia; Thus it is conceivable that these two variants might have an additional modifier effect on the disease. Further functional studies are necessary to substantiate this claim.

## Background

Multicentric carpotarsal osteolysis syndrome (MCTO), OMIM # 166300 is an extremely rare skeletal disorder (the frequency is not known), usually presenting in early childhood characterized by symptoms mimicking juvenile rheumatoid arthritis, and progressive bone loss of hands and feet, typically carpal and tarsal bones. Other symptoms include peculiar facial manifestations such as triangular shape of the face, micrognatia, protruding eyes, as well as nephropathy and eventual renal failure (Kohler et al., 1973; Tyler and Rosenbaum, 1976; Whyte et al., 1978; Lemaitre et al., 1983; Hardegger et al., 1985; Carnevale et al., 1987; Pai and Macpherson, 1988; Thomas et al., 2006).

According to https://rarediseases.info.nih.gov between 80 and 99% of all patients with MCTO have the following symptoms: cachexia, carpal osteolysis, EMG abnormality, abnormal gait, decreased joint mobility, metacarpal osteolysis, micrognathia, proptosis, proteinuria, slender long bone, triangular face, wrist swelling. Furthermore, 30–79% of patients have camptodactyly of finger, and nephropathy. Nephropathy in majority of cases is recognized lately during the course of the disease and leads to end stage renal failure. It manifests with proteinuria (Mehawej et al., 2013; Mumm et al., 2014); there are only few cases documented with renal biopsy, which disclosed focal segmental glomerulosclerosis (Hirooka and Hirota, 1985). In the French series two out of eight patients presented with bilateral kidney hypoplasia (Mehawej et al., 2013). Our patient had hypoplastic right kidney, but no significant proteinuria was detected at the last check-up.

Multicentric carpotarsal osteolysis syndrome is inherited in an autosomal dominant manner with a complete penetrance, and in most cases it occurs sporadically. It is caused by mutations clustering in the N-terminal activation of MAFB (Zankl et al., 2012; Mehawej et al., 2013; Mumm et al., 2014). MAFB is thought to cause imbalance in bone remodeling followed by osteolysis, likely resulting in suppression of RANKL/RANK signaling induced genes, which are crucial for osteoclastogenesis (Kim et al., 2007). Therefore, defects in the MAFB protein regulate RANKL-induced osteoclastogenesis in a negative manner, and collectively manifest as the abnormalities seen in MCTO patients. *MAFB* gene is involved in the process of nephrogenesis as well, and it is expressed in podocytes; hence it might account for the structural and functional changes in the kidneys. According to current literature, all MCTO patients are genetically homogeneous (mutations are found in the *MAFB* gene), with moderate clinical variability, possibly due to modifier effects (Mehawej et al., 2013).

## Discussion

### Multicentric carpo-tarsal osteolysis—treatment options

Multicentric carpo-tarsal osteolysis is a very rare disorder, with tens of cases reported in literature worldwide. Herein, by applying trio CES we solved a case, which had been left undiagnosed for more than a decade. To the best of our knowledge this is the first genetically diagnosed and reported case of MCTO in the Balkans. The case described in this report illustrates the power of CES to interrupt the diagnostic odyssey of this family. Although the establishment of the correct diagnosis did not result in a direct causal treatment, some preventive measurements should be implemented in this patient such as: corrective bone surgery (Sun et al., 2016), regular monitoring of proteinuria and renoprotective treatment, as well as avoiding or careful administration of non-steroidal anti-inflammatory and other nephrotoxic drugs. In the same vein, clinicians should take into account the therapeutic success reported in a couple of studies for the spectrum of osteolytic diseases with bisphosphonates, cyclosporine A, tocilizumab (Connor et al., 2007; Lee et al., 2010; Nishikomori et al., 2015; Pichler et al., 2016).

### Multicentric carpo-tarsal osteolysis—larger cohorts are needed

Multicentric carpotarsal osteolysis syndrome is a disorder, where mutations cluster in a hotspot region of the MAFB protein. MCTO has been shown to be always caused by aberrant MAFB function albeit with clinical variability. This variability can emanate from numerous sources. Firstly, MAFB is a basic leucine zipper transcription factor that plays a role in the regulation of lineage specific hematopoiesis, and osteoclastogenesis. It is therefore conceivable that minor changes, even in the same hotspot regions of the protein can lead to nuanced but nonetheless differential regulatory, and consequently transcriptional effects that can explain the clinical variability. Secondly, additional genetic or epigenetic variations can have a modifier effect and lead to clinical variability in a seemingly well-defined “genetic homogeneity.” In this context, application of broad DNA tests in lieu of focused gene panels, in a sufficiently high cohort of patients can provide more insights about modifier effects, even in the cases where the penetrance seems to be complete. Finally, the functional role of *MAFB* and its pathogenic variants should be further studied via biochemical, genomic, transcriptomic and epigenetic approaches in cell culture, and model organisms.

In our opinion, in order to sneak a peek into the molecular peculiarities of the disease, a more centralized MCTO institutional body should be established. This primary task of this body should be to find, select and study already diagnosed or candidate MCTO patients under one umbrella in a controlled manner. Such an effort could aid the discovery of candidate targets in the RANKL-associated pathway for development of precision therapies.

## Concluding remarks

The plummeting prices of next-generation sequencing (NGS) technologies, have led to broad availability of comprehensive DNA tests such as whole human genome sequencing (WGS), whole exome sequencing (WES), and CES in everyday clinical settings (Shendure and Lieberman Aiden, 2012; Katsanis and Katsanis, 2013). These approaches of massively parallel sequencing, coupled with new and easy-to-use platforms for NGS data processing, analysis, filtering, and interpretation are setting the foundation for a new wave of quick and cheaper clinical diagnostics, and accumulation of knowledge, particularly valuable in the context of rare diseases (Stark et al., 2017). Singleton exome/genome sequencing can lead to a definitive diagnosis in many cases, but the modus operandi of preference in genetic diagnostics, in our opinion should be trio or family cohorts, whenever possible.

Clinicians should embrace these new technologies and join efforts with geneticists in tackling and solving numerous rare disease cases. Ultimately, comprehensive DNA testing can be used to provide new insights of molecular pathways and switches and aid the development of new therapies.

## Ethics statement

Written and signed informed consent was obtained from all subjects or their legal guardians of this case study.

## Data access

Fastq data have been deposited to the SRA under the accession number SRP136097.

## Author contributions

GK: conceived and designed this case study; GK: processed, analyzed, and interpreted the clinical exome data with the help of AS, SM, MS, KJ; GK and SM: contributed to genetic counseling; VT, NN, and GZ: contributed in the assessment and interpretation of the clinical symptoms and data; IK and ZM: contributed in the recruitment of patients in the hospital and contributed intellectually; GK: wrote the manuscript with help in the clinical part from VT. All authors contributed to improvement of the manuscript and read the final version of the manuscript.

### Conflict of interest statement

GK, IK, and AS are employees of Bio Engineering LLC. The other authors declare that the research was conducted in the absence of any commercial or financial relationships that could be construed as a potential conflict of interest.
