# Supplementary material for: Trio Clinical Exome Sequencing in a Patient With Multicentric Carpotarsal Osteolysis Syndrome: First Case Report in the Balkans
Source: Front Genet. 2018 Apr 5;9:113. doi: 10.3389/fgene.2018.00113 (PMC5895704; doi:10.3389/fgene.2018.00113)
Supplement: Supplementary file 1 [file Presentation1.PDF]

Trio Clinical Exome Sequencing in a Patient with Multicentric Carpotarsal Osteolysis Syndrome: First Case Report in the Balkans

Aleksandra Stajkovska[2], Sanja Mehandziska[1], Margarita Stavrevska[2], Kristina Jakovleva[1], Natasha Nikchevska[1], Zan Mitrev[1], Ivan Kungulovski[2], Gjorgje Zafiroski[4], Velibor Tasic[3], Goran Kungulovski[2*]

[1]Zan Mitrev Clinic, Skopje, Republic of Macedonia; [2]Bio Engineering LLC, Skopje, Republic of Macedonia, [3]Department of Pediatric Nephrology, University Children's Hospital, Medical Faculty of Skopje, Republic of Macedonia, [4]Neuromedica Hospital, Republic of Macedonia

**Corresponding author:*

Dr. Goran Kungulovski, Bio Engineering LLC, Laboratory of Genetics and Personalized Medicine, Zan Mitrev Clinic

Ivan Agovski 7-1, 1000, Skopje, Republic of Macedonia

Phone: +389 70 234280

goran@bioengineering.mk

goran.kungulovski@zmc.mk

Supplementary Material

A

B

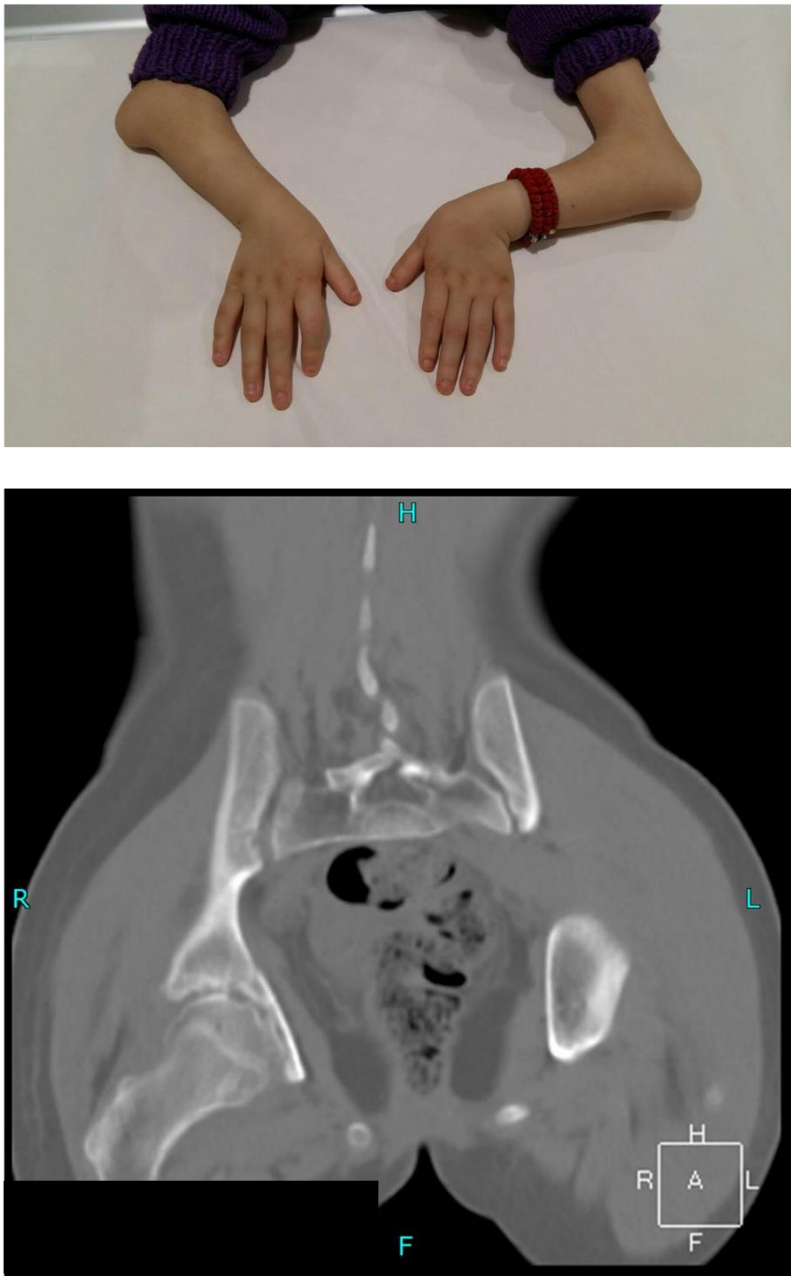

Supplementary Figure 1. Assessment of the clinical symptoms of the patient. A) Appearance of palms, hands and elbows of the patient. B) Computed tomography scan (CT) showing spina bifida and cyst-like structures on the right femoral epiphysis due to osteolysis.

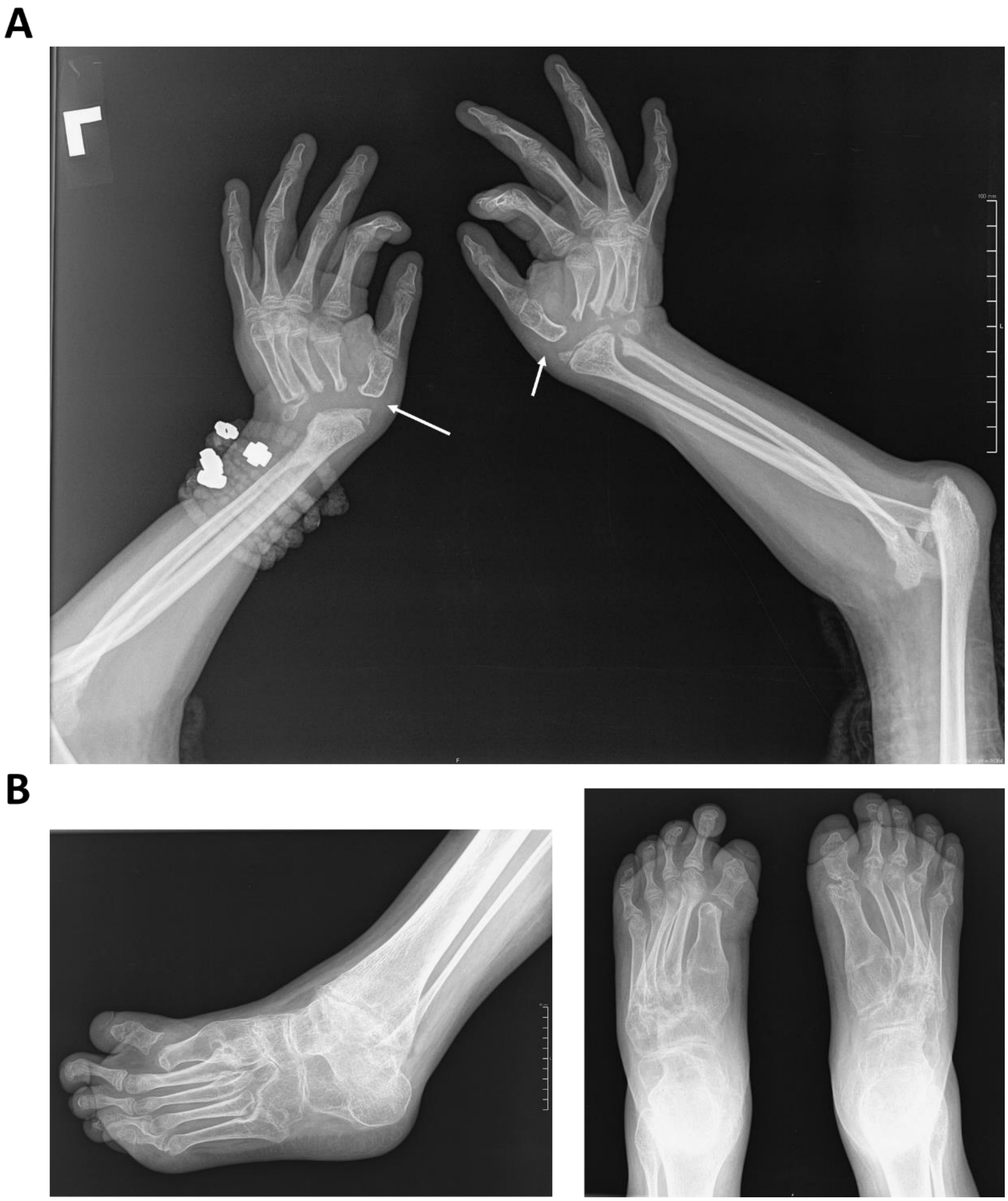

Supplementary Figure 2. Assessment of the clinical symptoms of the patient. A) X-ray image illustrating destruction of carpal bones. Absence of carpal bones, osteolysis of proximal end of metacarpal bones, and bones of the elbow joints with pathological dislocation of the elbow joints. B) X-ray image of feet illustrating tarsal osteolysis and metatarsals primus with pathological dislocation of the metatarsophalageal joint of both halluxes.

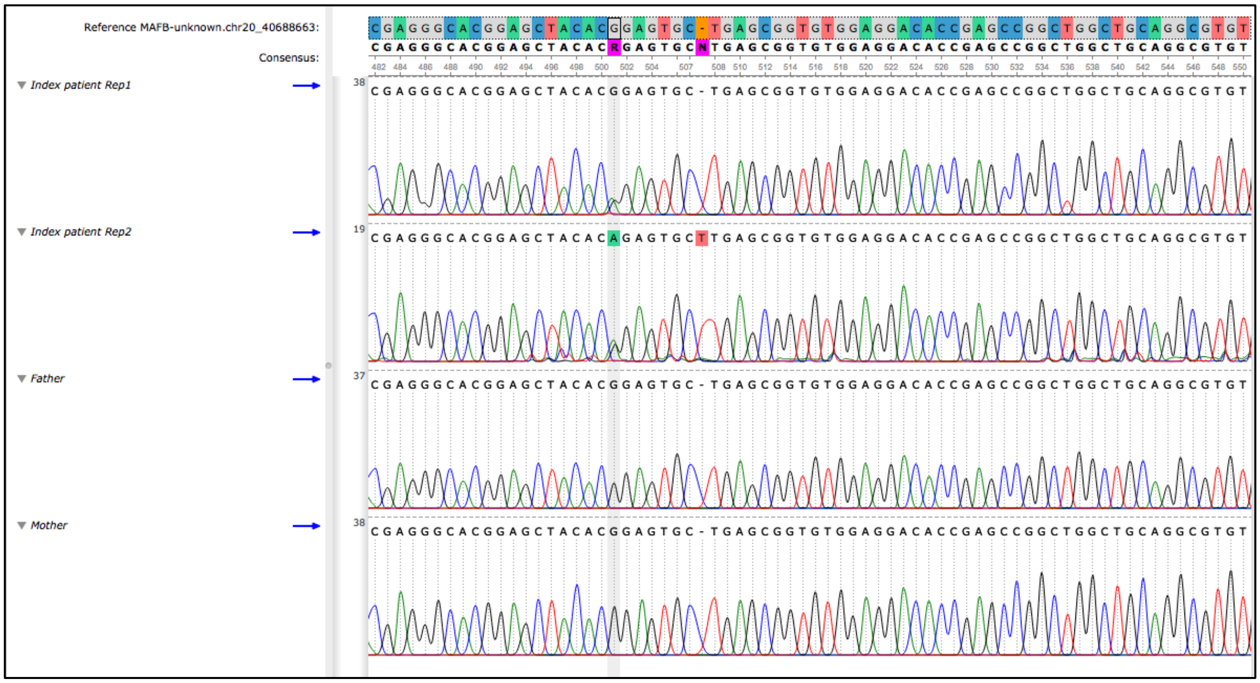

Supplementary Figure 3. Sanger sequencing of *MAFB* gene. Confirmation of c.188C>T (p.Pro63Leu) in the *MAFB* gene. Heterozygous presence of the c.188C>T (herein G>A) variant was confirmed in the patient, while no mutation (CC genotype) was detected in her mother and father.

Supplementary Table 1. A list of heterozygous candidate variants based on ACMG computational criteria found by singleton clinical exome sequencing.

Carriers	Gene	Type	Coding consequence	Chrom.	Genomic position	Coverage (proband)	Zygosity	c.DNA	Protein	dbSNP	g1000	esp5400	id_clinvar
Proband	*MAFB*	SNP	missense	20	39317302	168	Het	c.188C>T	p.Pro63Leu	/	/	/	/
Proband/Father	*COL9A3*	SNP	missense	20	61470100	75	Het	c.1851C>A	p.Asp617Glu	rs199577452	0.0002	0.00005	
Proband/Mother	*MATN3*	SNP	missense	2	20202930	108	Het	c.908C>T	p.Thr303Met	rs77245812	0.014	0.0161	rs77245812
Proband/Father	*COL10A1*	SNP	missense	6	116442542	263	Het	c.737T>C	p.Ile246Thr	rs764388204	0.00005	/	
Proband/Mother	*COL11A1*	SNP	missense	1	103453270	170	Het	c.2304A>T	p.Arg768Ser	rs367824632	/	0.0002	
Proband/Father	*COL11A1*	SNP	missense	1	103491461	45	Het	c.828A>C	p.Lys276Asn	rs12731843	0.0793	0.0712	
Proband/Father	*COL18A1*	SNP	missense	21	46875961	21	Het	c.517G>A	p.Gly173Ser	rs62000960	0.0016	0.0031	rs62000960
Proband/Father	*SMAD1*	SNP	missense	4	146460967	43	Het	c.412G>T	p.Val138Leu	rs371393906	/	0.00002	
Proband/Father	*LTBP1*	SNP	missense	2	33500098	120	Het	c.2810G>A	p.Ser937Asn	rs752887751	/	0.00008	
Proband/Father	*BMP2K*	SNP	missense	4	79831772	23	Het	c.2071G>A	p.Glu691Lys	rs182929460	0.0018	0.0048	
Proband/Father	*FLNA*	SNP	missense	X	153578543	211	Het	c.7189A>G	p.Asn2397Asp		/	/	
Proband/Father	*GC*	SNP	no-start	4	72669661	124	Het	c.3G>T	p.Met1?	rs76781122	0.0134	0.0172	

Supplementary Table 2. The c.188C>T variant in the MAFB gene meets numerous computational criteria of pathogenicity.

Carriers	Gene	Type	Coding consequence	Chrom.	Genomic position	Coverage (proband)	Zygosity	c.DNA	Protein	dbSNP	g1000	GERP	DANN	Mutation Taster	SIFT	FATHMM	MetaSVM
Proband	*MAFB*	SNP	missense	20	39317302	168	Het	c.188C>T	p.Pro63Leu	/	/	4.61	0.9982	Damaging	Damaging	Damaging	Damaging
